# In Vitro Antigenotoxic, Antihelminthic and Antioxidant Potentials Based on the Extracted Metabolites from Lichen, *Candelariella vitellina*

**DOI:** 10.3390/pharmaceutics12050477

**Published:** 2020-05-24

**Authors:** Islam El-Garawani, Mahmoud Emam, Waill Elkhateeb, Hesham El-Seedi, Shaden Khalifa, Salwa Oshiba, Shaimaa Abou-Ghanima, Ghoson Daba

**Affiliations:** 1 Department of Zoology, Faculty of Science, Menoufia University, Menoufia 32511, Egypt; 2 College of Pharmaceutical Science & Collaborative Innovation Center of Yangtze River Delta Region Green Pharmaceuticals, Zhejiang University of Technology, Hangzhou 310014, China; me.hegazy@nrc.sci.eg; 3 Phytochemistry and Plant Systematics Department, National Research Centre, 33 El Bohouth St., Dokki, Giza 12622, Egypt; 4 Chemistry of Natural and Microbial Products Department, Pharmaceutical Industries Researches Division, National Research Centre, Dokki, Giza 12311, Egypt; wm.el-khateeb@nrc.sci.eg (W.E.); gm.daba@nrc.sci.eg (G.D.); 5 Department of Molecular Biosciences, The Wenner-Gren Institute, Stockholm University, S-10691 Stockholm, Sweden; shaden.khalifa@su.se; 6 International Research Center for Food Nutrition and Safety, Jiangsu University, Zhenjiang 212013, China; 7 Al-Rayan Research and Innovation Center, Al-Rayan Colleges, Medina 42541, Saudi Arabia; 8 Pharmacognosy Group, Department of Medicinal Chemistry, Uppsala University, 75 123 Uppsala, Sweden; 9 Parasitology Department, Faculty of Medicine, Menoufia University, Menoufia 32511, Egypt; salwa.oshaiba@med.menofia.edu.eg; 10 Biochemistry Department, King Khalid University, Abha 61321, Saudi Arabia; sjamal@kku.edu.sa

**Keywords:** antihelminthic, antioxidant, apoptosis, *Candelariella vitellina* metabolites, DNA damage, HPLC, GC-MS

## Abstract

Lichens have recently received great attention due to their pharmacological potentials. The antigenotoxic potential of *C. vitellina* extract (25 and 50 µg/mL) was assessed in normal human peripheral blood lymphocytes (HPBL) against Mitomycin C (MMC) co-treatments. Flow cytometric analyses of cell cycle distribution, as well as apoptosis (Annexin V/PI), revealed that the extract had significantly (*p* ≤ 0.05) ameliorated the MMC toxicity by reducing the apoptotic cells and normalized the cell cycle phases. *C. vitellina* exhibited antigenotoxicity by ameliorating the diminished mitotic index and DNA single-strand breaks caused by MMC. Herein, the hydromethanolic extract (80%) of *Candelariella vitellina* (Japan) lichen, exhibited very low cytotoxicity towards normal human peripheral lymphocytes (HPBL) with IC_50_ >1000 µg/mL. In order to explore the antihelminthic effect, *Echinococcus granulosus* protoscoleces were used in vitro. Eosin staining revealed significant (*p* ≤ 0.05) dose and time-dependent scolicidal effects of the extract confirmed by degenerative alterations as observed by electron scan microscopy. Furthermore, primary and secondary metabolites were investigated using GC-MS and qualitative HPLC, revealing the presence of sugars, alcohols, different phenolic acids and light flavonoids. Significant antioxidant capacities were also demonstrated by DPPH radical-scavenging assay. In conclusion, the promising antigenotoxic, antihelminthic and antioxidant potentials of *C. vitellina* extract encourage further studies to evaluate its possible therapeutic potency.

## 1. Introduction

Lichens are naturally produced from the symbiotic associations between algae and fungi [1]. Compounds originating from lichen extracts have attracted attention due to their promising biopharmaceutical activities such as antiulcerogenic, anti-inflammatory, anticancer, immunostimulating, antioxidant, antipyretic, analgesic, and antimicrobial capabilities [2,3,4,5,6]. However, few studies were reported on the genotoxic as well as antigenotoxic activities of lichens. *In vitro* and in vivo studies on lichens extracts revealed that they exhibited antigenotoxic and non-genotoxic activities at the same time such as *Cetraria* species (*C. aculeata; C. islandica;* and *C. olivetorum*); three *Cladonia* species (*C. chlorophaea; C. foliacea;* and *C. rangiformis*); two species of *Peltigera* (*P. rufescens;* and *P. canina*). The same was applied to extracts of *Dermatocarpon intestiniforme; Parmelia pulla; Pseudevernia furfuracea; Ramalina capitata; and Xanthoria elegans* [7].

The first study to evaluate the genotoxic activity of the lichens secondary metabolites, physodic, and physodalic acids, was conducted in 1984 [8]. The non-genotoxic effect of (+)-usnic acid and (−)-usnic acid enantiomers extracted from the lichens *Ramalina farinacea* and *Cladonia foliacea*, respectively, has been also studied [9]. The antiproliferative activities of usnic acid and other lichens’ metabolites were also reported [3,10,11]. The protective potential of numerous lichen metabolites/extracts against DNA damage is stimulated by compounds such as aflatoxin B_1_, methyl nitro-nitrosoguanidine, methyl methane-sulfonate and colloidal bismuth subcitrate was reported [12,13,14,15,16,17].

Free radicals and oxidants cause oxidative stress when they exist at high levels. Oxidative stress, if not regulated properly, can cause severe and chronic diseases such as Alzheimer’s disease, Parkinson’s disease, atherosclerosis, emphysema, schizophrenia, hemochromatosis, cancer, and cells aging [7,18]. Antioxidants are compounds capable of protecting the body from the damage induced by oxidative stress. Extracts of lichens such as *Ramalina conduplicans*, *Lasallia pustulata* and *Peltigera laciniata* [19,20,21] are rich in phenolic metabolites such as depsidones, depsides, pulvinic acid derivatives, and dibenzofurans that possess antioxidant properties [22].

*Candelariella vitellina* is a lichen that inhabits trees and woods. The in vitro and in vivo anticancer potentials of *C. vitellina* have been described in a previous study of El-Garawani et al. [23], where *C. vitellina* extract exerted promising antioxidant and pro-apoptotic activities on Caco-2 cells and Ehrlich solid tumor. Moreover, seven novel compounds were detected, and 11 compounds were identified as terpenes and polyketides [23]. The in vitro anti-colorectal cancer, hypocholesterolemic, anti-rota virus activities of *C. vitellina* extract have been also reported [24].

Cytotoxic medications, belonging to various schemes of cancer treatments, are an important intervention to combat cancers [25]. However, the majority of chemotherapeutic agents cause adverse effects such as genotoxic, carcinogenic and teratogenic impacts in vivo and in vitro [26], owing to the genetic alterations produced by their interactions with specific biological molecules in normal cells followed by the generation of free radicals [27]. Mitomycin C (MMC), a bifunctional alkylating agent isolated from *Streptomyces caespitosus*, is one of these applied antitumor drugs [28]. MMC causes DNA-cross linkage leading to genotoxicity and cancer by superoxides and hydroxyl radicals’ production [29,30]. It induces genotoxic and oxidative DNA damages, single/double DNA-strand breaks and apoptosis [31,32]. Chromosomal aberrations, sister chromatid exchanges and elevated micronucleus frequency in human lymphocytes were also reported [33]. Applying natural extracts enriched with antioxidants may facilitate the repair processes or neutralize the damaging factors that affect DNA molecules [34,35]. Besides, they can reduce the genotoxic effects and modulate the mechanisms of the organism’s defense by which it can avoid the incidences of cancer and other mutation-related diseases.

The helminth parasites in humans are a devastating public health problem in endemic areas. Various chemicals including 20% hypertonic saline, Ag-nitrate, and cetrimide have been applied as antihelminthic agents, but they have shown severe complications [36,37]. The principal antihelminthic chemotherapeutics used for echinococcosis are benzimidazole derivatives such as albendazole (ALB) and mebendazole. They inhibit the polymerization of microtubules by selective binding to parasite tubulin [38].

Cystic echinococcosis (CE) or hydatidosis is a considerably infectious disease with severe health hazards. It is a zoonotic parasitic infection caused by *Echinococcus granulosus*. The life cycle of *E. granulosus* includes dogs and wild carnivores as a definitive host, and sheep, cattle, camel and goats as intermediate hosts. Humans can also act as an intermediate host for *E. granulosus* and the infection occurs through the ingestion of parasite eggs in contaminated food, water, soil or direct contact with infected animals [39]. It is considered a public health problem, especially in developing countries [40,41].

Several herbal extracts showed promising potent scolicidal and antischistosomal effects [42,43]. The herbs’ protective potentials against various toxic agents [44,45,46,47], anticancer activities towards many types of cancers and anti-inflammatory properties [48,49,50,51,52,53,54] were also reported in vivo and in vitro.

Taken together, studies on the biological potentials of this promising lichen are still insufficient. In this study, and based on the phytochemical investigated metabolites, the hydromethanolic extract of *Candelariella vitellina* was investigated for its in vitro antigenotoxic, antihelminthic and antioxidant potentials. To the best of our knowledge, this study is the first to investigate the antigenotoxic and antihelminthic potentials of the *C. vitellina* lichen.

## 2. Materials and Methods

### 2.1. Materials

#### 2.1.1. Lichen Material

In continuation of the previous work of El-Garawani et al. [23], lichen was collected from the barks of the trees (Hakozaki, Higashi-ku, Fukuoka-shi, Japan) and identified as *Candelariella vitellina*. A stock solution of 1:1 (*v/v*) hydromethanolic extract (80%) of *C. vitellina* in DMSO was prepared and kept at −4 °C for further biological investigations.

#### 2.1.2. Genotoxic Drug

Mitomycin C, MMC, (C_15_H_18_N_4_O_5_) (Lyomit, mitomycin C kyowa, Biochem, India) was enrolled in this study. A stock solution of MMC was prepared in distilled water under sterile conditions. It was applied (co-treatment) as a genotoxic agent with final concentrations of 0.5 µg/mL (19 mg/m^2^) in the culture medium. The dose was within the higher therapeutic range.

#### 2.1.3. Antihelminthic Drug

Albendazole, ALB, (C_12_H_15_N_3_O_2_S) is an antihelminthic drug with broad-spectrum applications (Bendax^®^, SIGMA pharmaceuticals, Monufia, Egypt). A stock solution of 1:1 (*v/v*) ALB in DMSO was prepared. It was used in this study as a positive control with a final concentration of 50 μg/mL in culture medium.

### 2.2. Methods

#### 2.2.1. Phytochemical Analyses

##### Preparation of the Extract

The extract was prepared by soaking about 100 g of the lichen in 10× (*w/v*) of methanol (80%) for 12 h at 4 °C The mixture was then filtered and concentrated to dryness under reduced pressure using a rotary evaporator (Rotavapor^®^, Heidolph, Schwabach, Germany) at 40–45 °C. The dried extract was refrigerated until used for further investigation at −4 °C [50].

##### Estimation of Total Phenolic and Flavonoid Contents

The total phenolic content (TPC) was determined according to the Folin–Ciocalteu procedure [55] and constructing a calibration curve. A serial dilution of gallic acid (5–50 μg) was prepared and measured at 725 nm. The linear equation of Y = 0.024X + 0.018 with a regression coefficient (R^2^) = 0.998, the plot with a slope (m) = 0.024 and intercept = 0.018 was used to determine the phenolic contents of the *C. vitellina* extract. As well, the total flavonoid content (TFC) of the total hydroalcoholic extract was determined according to Žilić et al. [55] using aluminum chloride (AlCl_3_) colorimetric assay and catechin as standard. The catechin solution of concentration (2.5–25 μg) conformed to Beer’s Law at 510 nm with a regression coefficient (R^2^) = 0.998. The plot has a slope (m) = 0.008 and intercept = 0.012. The equation of the standard curve was Y = 0.012X + 0.008.

##### Preliminary Qualitative Phenolic Analysis of the Extract

Different tests were used to determine the classification of phenolics present in the *C. vitellina* extract as Shinoda’s test for flavonoids [56], Ferric chloride test for phenolics [57], Potassium Iodate KIO_3_ test specific for galloyl esters spraying reagent [58] and sodium nitrite (NaNO_2_) assay for ellagitannins [59,60].

##### GC-MS Analysis

The GC-MS analysis of the sample was performed at Department of Medicinal and Aromatic Plants Research, National Research Center (Dokki, Giza, Egypt) with the following specifications: a TRACE ™ 1300 GC Ultra Gas Chromatograph (Thermo Fisher Scientific, Austin, TX, USA), coupled with a thermo mass spectrometer detector (ISQ Single Quadrupole Mass Spectrometer). The GC-MS system was equipped with a TR-5 MS column (30 m × 0.32 mm i.d., 0.25 μm film thickness). Analyses were carried out using helium as the carrier gas at a flow rate of 1.0 mL/min and a split ratio of 1:10 using the following temperature program: 60 °C for 1 min, rising at 4.0 °C/min to 240 °C and held for 1 min. Both injector and detector were held at 210 °C. Mass spectra were obtained by electron ionization (EI) at 70 eV, using a spectral range of *m/z* 40–450.

##### GC-MS Analysis of Silylated Metabolites

Metabolite analysis was carried out as follows. Briefly, a sample of *C. vitellina* extract (100 mg) was extracted with 5 mL HPLC grade methanol using ultrasonic bath for 30 min with repeated shaking, followed by centrifugation at 10,000 rpm for 10 min. Aliquots of the methanolic extract (150 µL) were kept in screw-cap vials and left to evaporate under a nitrogen gas stream until completely dry. For derivatization, 150 µL of *N*-methyl-*N*-(trimethylsilyl)-tri fluoroacetamide (MSTFA) that was previously diluted 1:1% with anhydrous pyridine was added to the dried methanolic extract and incubated at 60 °C for 45 min prior to GC-MS analysis [61].

##### Qualitative Determination of Polyphenols Using HPLC Analysis

HPLC analysis was carried out using an Agilent 1260 series, USA. The separation was carried out using a Kromasil C18 column (4.6 mm × 250 mm i.d., 5 μm). The mobile phase consisted of water (A) and 0.05% trifluoroacetic acid in acetonitrile (B) at a flow rate of 1 mL/min. The mobile phase was programmed consecutively in a linear gradient as follows: 0 min (82% A); 0–5 min (80% A); 5–8 min (60% A); 8–12 min (60% A); 12–15 min (85% A) and 15–16 min (82% A). The multi-wavelength detector was monitored at 280 nm. The injection volume was 10 μL for each of the sample solutions. The column temperature was maintained at 35 °C.

##### Antioxidant (Radical Scavenging Activity)

The free radical scavenging activity of *C. vitellina* extract was measured by 2, 2-diphenyl-1-picryl-hydrazil (DPPH^•^) using both methods of Shimada et al. [62] and Hwang and Do Thi [63]. EC_50_ values were calculated for the most active extracts possessing ≥90% scavenging activity using probit analysis and utilizing the SPSS computer program (SPSS for windows, statistical analysis software package/version 9/1989 SPSS Inc., Chicago, IL, USA) depending on the DPPH^•^ scavenging effect (%) = 100 − [((A0 − A1)/A0) × 100], where A_0_ was the absorbance of the control reaction and A_1_ was the absorbance in the presence of the sample [64]. The free radical scavenging capacity of the extract was determined using the stable DPPH^•^ according to Hwang and Do Thi and the standard curve was prepared using Trolox. Results were expressed as mg Trolox equivalents (TE)/g sample). Additional dilution was needed if the DPPH^•^ value measured was over the linear range of the standard [63].

#### 2.2.2. Cytotoxicity Studies on Normal Human Peripheral Blood Lymphocytes (HPBL)

##### HPBL Proliferation Assay (MTT Assay)

The mononuclear cells of peripheral blood (EDTA-anticoagulated), from three non-smoker and healthy male volunteers, were isolated by density gradient centrifugation method at 1500 rpm for 10 min through the Ficoll-Hypaque (Lonza, Basel, Switzerland). The study was approved by the Ethics Committee at the Faculty of Science, Menoufia University (MUFS-GE-1-20) after obtaining written consent from all participants. Using a trypan blue (0.4%) exclusion technique, the cells’ viability was calculated using a hemocytometer. Samples of less than 95 percent of viable cells and more than 2 percent of red blood cells contamination were excluded. For performing MTT assay, about 2 × 10^4^ of isolated mononuclear cells in complete RPMI (RPMI-1640 medium, 15% FBS and 1% penicillin/streptomycin) supplemented with phytohemagglutinin-L (Sigma-Aldrich, St. Louis, MO, USA) were incubated in a 96-well microtiter plate for 48 h. Thereafter, serial concentrations of *C. vitellina* were applied in triplicate and the cultures were extended for another 24 h. After the incubation period, MTT (5 mg/mL) was added to each well for 4 h at 37 °C. Then, the resulting formazan crystals were dissolved using 0.04 N HCl in isopropanol and the absorbance was recorded at 570 nm using a microplate reader (RADIM SEAC Sirio S, Rome, Italy). 

All performed procedures in this study including human participants were in accordance with the ethical standards of the institutional research committee at Faculty of Science, Menoufia University, Egypt (MUFS-F-GE-1-20) and with agreement to the 1964 Helsinki declaration and its later amendments.

##### Cell Culture and Isolation

The extract toxicity was tested on HPBL isolated from the same volunteers mentioned above (MTT assay). Peripheral venous samples of blood were collected using sterile syringes and then transferred into sterile tubes (KEMICO vacutainer, Cairo, Egypt) containing anticoagulant. Samples were processed for culturing in RPMI-1640 medium supplemented with 15% fetal bovine serum, 2% phytohemagglutinin and 1% (100 U/mL penicillin and 100 μg/mL streptomycin) at 37 °C and humidified 5% CO_2_ atmosphere (1 whole blood: 4 complete media, *v/v*). After 48 h of culture setup, various treatments (Untreated, DMSO, MMC, MMC co-treatments with 25 and 50 μg/mL of the extract) were applied and the cultures extended for other 24 h. For HPBL isolation after the treatment period, cultures were incubated with three folds of erythrocyte lysing buffer (0.015M NH_4_C1, 1 mM NaHCO_3_, 0.l mM EDTA) for 10 min at 37 °C. Then, centrifugation was done for 3 min at 1000 rpm. The incubation was repeated until a pellet of lymphocytes appeared [47]. All reagents were from Lonza, Switzerland.

##### Acridine Orange/Ethidium Bromide (AO/EB) Dual Fluorescent Staining

The viability of cells was quantified using a fluorescence microscope (Olympus BX 41, Tokyo, Japan) using AO/EB double staining. Briefly, 4 μL of treated and control cells’ suspension were stained with 1 μL stain solution AO/EB (100 μg/mL AO and 100 μg/mL EB) on glass slides and examined immediately at 400× magnification. Randomly four fields were observed and 300 cells were counted from each. Two types of cells were observed, based on the emitted fluorescence: viable cells were green-colored cells with intact structures and late apoptotic or dead cells showed an orange-to-red color [65].

##### Quantification of Apoptosis Using Annexin V/PI Labeling

Apoptosis and necrosis were assessed using flow cytometry following the instruction manual of the Annexin V-FITC Kit (BD Pharmingen™, San Diego, CA, USA). After various treatments, HPBL were then trypsinized, rinsed twice with PBS, labeled with FITC-conjugated Annexin V antibody and stained with propidium iodide (Invitrogen, Carlsbad, CA, USA). Samples were analyzed using BD Accuri™ C6 flow cytometer, US.

##### Flow Cytometric Analysis of the Cell Cycle

The effect of *C. vitellina* on the cell cycle phases of treated HPBL was analyzed using flow cytometry. Following 24 h of different treatments, cells were digested with trypsin (1×) and washed with ice-cold PBS, fixed in ethanol and labeled with PBS containing propidium iodide (1 mg/mL) and RNase A (200 μg/mL) for 10 min. The cells’ percentage in sub-G_1_, G_0_/G_1_, S, or G_2_/M phases was evaluated using DNA analysis program MODFIT (Verity Software House, Topsham, ME, USA, version: 2.0). Cell cycle analysis was performed using BD Accuri™ C6 flow cytometer, San Jose, CA, USA.

#### 2.2.3. Genotoxicity Studies on Normal Human Peripheral Blood Lymphocytes (HPBL)

##### Mitotic Index

Chromosomal preparation was performed following the method of Evans (1976) [66] to assess the mitotic index of control and treated HPBL. Colcemid (10 µg/ml) was incubated with the cells for 2 h to arrest the division, then cells were harvested and resuspended in a hypotonic solution (0.4% KCl) for 20 min at 37 °C. The pellets of cells were fixed in freshly-prepared 3:1 (*v/v*) methanol: glacial acetic acid. Fixed cells were resuspended in 100 µL of the fixative and dropped onto glass slides. Air-dried cells were stained with 3% (*w/v*) Giemsa in phosphate buffer. For mitotic evaluation, about 500 cells of each culture were examined by an Olympus BX41 (Tokyo, Japan) light microscope at the magnification of 200×. The mitotic index was calculated according to the following equation:

Mitotic index (%) = (metaphases + prophases) × 100/(metaphases + prophases + non dividing cells)(1)

##### Comet Assay

For evaluating the DNA single-strand breaks, alkaline comet assay was performed according to Singh et al. (1988) [67]. Following the treatment period, HPBL were harvested and about 3 × 10^4^ cells were embedded in a pre-heated 0.5% low melting point agarose (Sigma-Aldrich, Darmstadt, Germany) in PBS. Cells’ suspension was layered between two layers of 0.7% ultra-pure normal point melting agarose (Sigma-Aldrich, Darmstadt, Germany) on the pre-coated slides with 1% normal melting point agarose. Subsequently, slides were immersed in lysis buffer (2.5 M NaCl, 100 mM EDTA, 10 mM Tris, 1% Triton X-100, and 10% DMSO) at 4 °C for 1 h. The slides were then incubated in the electrophoresis tank in an alkaline (pH > 13) electrophoresis buffer (300 mM NaOH and 1 mM EDTA) for 20 min to facilitate the unwinding of DNA before carrying out the electrophoresis process. After electrophoresis, the slides were neutralized with 0.4 M Tris-HCl (pH 7.5) buffer and washed with PBS then stained with ethidium bromide (20 µg/mL). Finally, 200 cells per slide were evaluated using an Olympus BX 41 UV-fluorescence microscope (Tokyo, Japan).

#### 2.2.4. Antihelminthic Studies

##### Collection of *Echinococcus granulosus* Protoscoleces

The protoscoleces of *E. granulosus* were acquired from the lungs of naturally infected sheep slaughtered at Shebin El-Kom Slaughter House (Menoufia Governorate, Shebin El-Kom, Egypt). The hydatid fluids were aspirated by a 20 mL syringe and conveyed into a bowl, then left to set for 30 min for protoscoleces precipitation. Then, they were centrifuged at 800 rpm for 3 min and washed two times with PBS. Protoscoleces had a length of approximately 0.3–0.4 mm. The number of protoscoleces per mL was adjusted as 2 × 10^3^ protoscoleces in 0.9% NaCl solution. The viability of the protoscoleces was established by their flame cell motility and impermeability to 0.1% eosin stain under a light microscope (Olympus BX41, Japan) [68,69].

##### Protoscoleces Maintenance and Treatments

To explore the therapeutic scolicidal effects of *C. vitellina* extract against protoscoleces of hydatid cysts, four concentrations of the extract (100, 300, 500 and 1000 μg/mL), 50 μg/mL of albendazole (ALB), DMSO and untreated control were used. Briefly, 2 × 10^3^ /mL of the protoscoleces were incubated in RPMI 1640 medium at 37 °C for 0.5, 1.5 and 3 h. At the end of each incubation time, the media were carefully discarded then set, to define the viability of protoscoleces.

##### Determination of Protoscoleces Viability

To evaluate the viability of protoscoleces, a 0.1% eosin solution was mixed with protoscoleces in a ratio of 1:1 and incubated for 5 min. The settled pellet of protoscoleces was then smeared on a glass slide, covered with a cover glass and examined under the light microscope (Olympus BX41, Tokyo, Japan). The percentages of dead protoscoleces were evaluated by counting 300 protoscoleces in at least three microscopic fields. Dead protoscoleces absorbed eosin and appeared with red color, while live protoscoleces stayed colorless and presented characteristic muscular movements with flame cell activity. All experiments were done in triplicate [70,71].

##### Scanning Electron Microscopy (SEM)

After 3 h of the incubation, parasites were processed for scanning electron microscopy at the Electron Microscopy Unit of Theodor Bilharz Research Institute (Giza, Egypt). Fixed specimens were washed in distilled water, treated with 1% uranyl acetate for 30 min. Subsequently, samples were washed lengthily with distilled water and dehydrated by incubation in sequentially increasing concentrations (50%, 70%, 80% and 90%) of ethanol. Samples were then washed in PBS (pH 7.2) and treated with 1% uranyl acetate for 30 min. Finally, they were coated, inspected and examined by a Joel JEM-1200 (Boston, MA, USA) for evaluating the morphological alterations [72].

#### 2.2.5. Data Analysis

The experiments were performed in triplicate. The data are represented as means ± standard deviations. The results of the statistical tests were determined by the *t*-test in Microsoft Excel. The value of *p* ≤ 0.05 was considered statistically significant.

## 3. Results

### 3.1. Phytochemical Analyses

#### 3.1.1. Estimation of Total Phenolic (TPC) and Flavonoid (TFC) Contents

The TPC and TFC of *C. vitellina* extract were quantitatively evaluated. The contents were estimated from the standard curves as 80.89 ± 0.27 (mg GAE/g) and 43.36 ± 0.038 (mg CE/g), respectively confirming the existence of phenolic structures.

#### 3.1.2. Preliminary Qualitative Analysis of the Extract

The constituents of *C. vitellina* extract were examined and showed mainly classes of phenolics and flavonoids as shown in Table 1. Gallotannins and ellagitannins were not detected. Moreover, two-dimensional paper chromatography (2D-PC) was performed and the results revealed the presence of phenolic compounds after spraying with different reagents. Corresponding spots gave positive responses towards FeCl_3_ spray reagent, indicating the presence of light flavonoid derivatives that appeared under short UV light as dark purple spots which turned into yellow when fumed with ammonia vapor, while the intense blue green color indicating the presence of phenolic compounds.

#### 3.1.3. GC-MS Studies of Silylated Metabolites

The prepared samples were injected into GC-MS and the major primary metabolites peaks were numbered and classified as sugars, alcohols, and acids (Table 2). As well, the identification of compounds depend on the Wiley spectral library collection and NIST library databases (www.amdis.net).

#### 3.1.4. Polyphenolics Analysis Using HPLC

Phenolic compounds of *C. vitellina* were detected using HPLC against 16 different standards of phenolic acids and flavonoids. The qualitative analysis depended on the retention time afforded to the presence of different phenolic acids and flavonoids (Table 3). The concentrations of the main eight identified metabolites were measured (Table 3).

#### 3.1.5. Antioxidant Activities

The total equivalent antioxidant capacity (TEAC) of *C. vitellina* extract using DPPH displayed good radical-scavenging activities with EC_50_ = 67.6 ± 0.7μg/mL and 92.01 ± 0.374 mg TE/g (trolox equivalent).

Depending on the visual observation of phenolic preliminary tests, especially the ferric chloride test, further quantitative and qualitative investigations of *C. vitellina* extract, the dihydroxy benzoic acid derivatives moieties were detected using GC-MS and HPLC. The identified phenolic acids were gallic, chlorogenic, syringic, pyrocatechol, protocatechuic, caffeic, and ellagic acids. In addition, rutin and taxifolin were also detected along with acids of primary metabolites such as lactic, acetic, lauric, myristic, palmitic, and stearic acids.

### 3.2. Protective Effect of C. vitellina Extract

#### 3.2.1. Cytotoxicity of *C. vitellina* on HPBL (MTT Assay)

**Figure 1:**
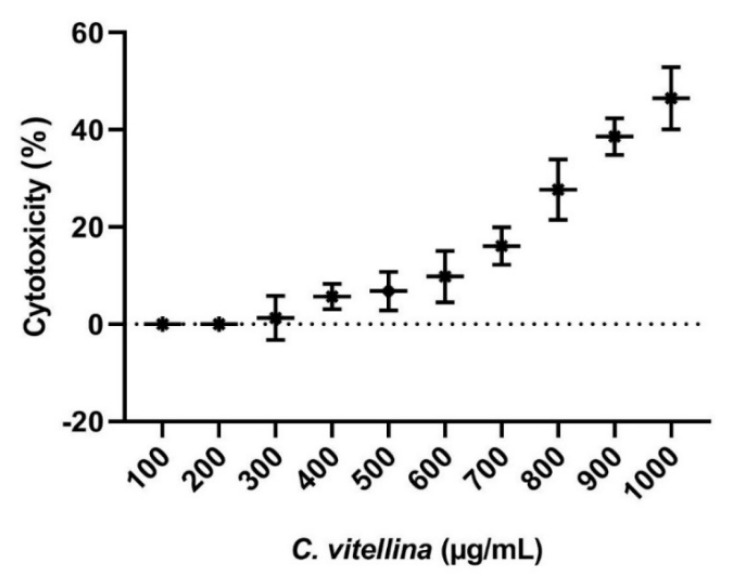
The effect of *C. vitellina* extract on HPBL cytotoxicity, 24 h, using MTT assay. Incubation with serial concentrations of the extract showed very weak toxicity with an IC_50_ > 1000 µg/mL. Data were represented as (Mean ± SD) of three different experiments (n = 3).

The cytotoxicity of *C. vitellina* extract was tested against HPBL using MTT assay. The value of IC_50_ was higher than 1000 µg/mL (Figure 1). This value indicated that *C. vitellina* exhibited very low cytotoxic potential towards normal HPBL. The extract showed no toxicity up to the concentration of 250 µg/mL.

#### 3.2.2. Assessment of Viability by AO/EB Double Fluorescent Staining

**Figure 2:**
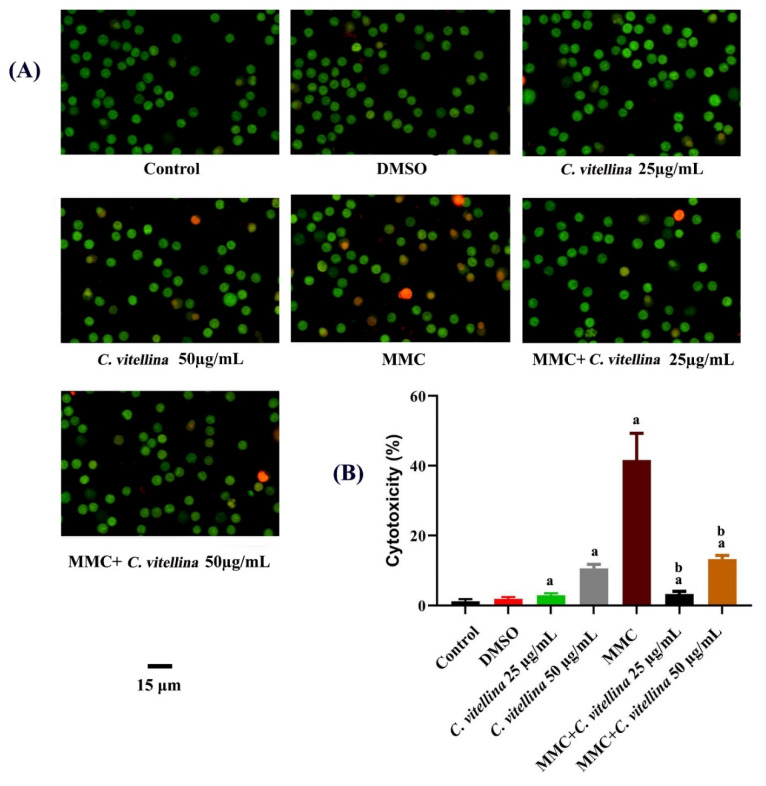
Photomicrographs (**A**) showing the significant ameliorative effect of *C. vitellina* extract on HPBL viability, 24 h against MMC toxicity, using acridine orange/ethidium bromide (AO/EB) dual fluorescent staining (Olympus BX 41 fluorescent microscope, Tokyo, Japan). Significant differences at (*p* ≤ 0.05) with respect to untreated and MMC-treated groups are (a) and (b), respectively. Data were represented as (Mean ± SD) of three different experiments (n = 3), (**B**). MMC: Mitomycin C (0.5 µg/mL).

The fluorescent AO/EB double staining was used to evaluate the viability of HPBL. The HPBL staining displayed uniform fluorescent green cells in the control and DMSO-treated groups, whereas apoptotic cells in the early stage were marked by the yellow-green color. Orange nuclei revealed necrotic and late-stage apoptotic cells among treated groups; especially in MMC-treated groups (Figure 2A). The significant (*p* ≤ 0.05) reduction in toxicity was observed among 25 and 50 µg/mL of *C. vitellina* extract (3.38 ± 0.76 and 13.23 ± 1.04, respectively) in comparison to MMC (41.67 ± 7.63) records (Figure 2B).

#### 3.2.3. Quantification of Apoptosis vs. Necrosis

**Figure 3:**
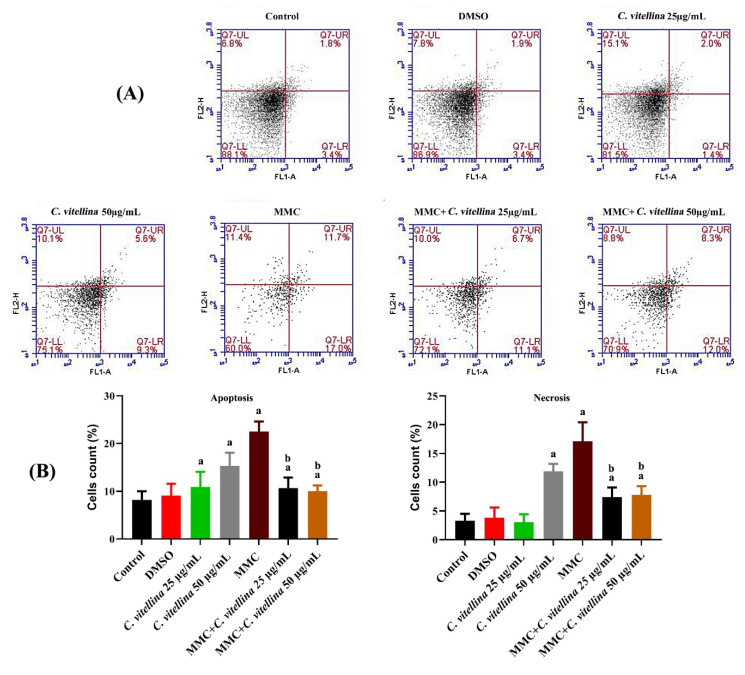
Representative histograms showing the ameliorative effect of *C. vitellina* extract on HPBL, 24 h against MMC toxicity, using Annexin V-FITC/PI assay showing the apoptotic stages and necrosis (**A**). The percentages of cells’ distribution of apoptosis and necrosis are illustrated in the histograms. (**B**). Results show the significant differences at (*p* ≤ 0.05) in co-treated groups with respect to untreated (a) and MMC-treated (b) groups. Data were represented as (Mean ± SD) of three different experiments (n = 3). MMC: Mitomycin C (0.5 µg/mL).

The flow cytometric analyses after Annexin-V FITC/PI labeling were performed to assess the protective effect of *C. vitellina* extract against MMC-induced cell death through apoptosis or necrosis in HPBL. Results are presented in Figure 3A,B, where the significant (*p* ≤ 0.05) anti-apoptotic (early and late) cells’ accumulation among the extract co-treated groups compared to the MMC-treated group was evidenced. The anti-apoptotic effect in the co-treated groups reached about 55% and 53.7% for 25 and 50 µg/mL of the extract respectively, when compared with MMC-treated cells. However, all treated groups showed significant (*p* ≤ 0.05) apoptotic populations (early and late) lesser than MMC group records. The results revealed a significant (*p* ≤ 0.05) elevation of necrotic events in all treated groups except the lower concentration of the extract (25 µg/mL). The significant (*p* ≤ 0.05) protective effect towards the necrotic properties in the extract co-treated groups was shown with up to 56.5% and 54% for 25 and 50 µg/mL of the extract respectively when compared with MMC-treated cells (Figure 3B).

#### 3.2.4. Cell Cycle Analysis

**Figure 4:**
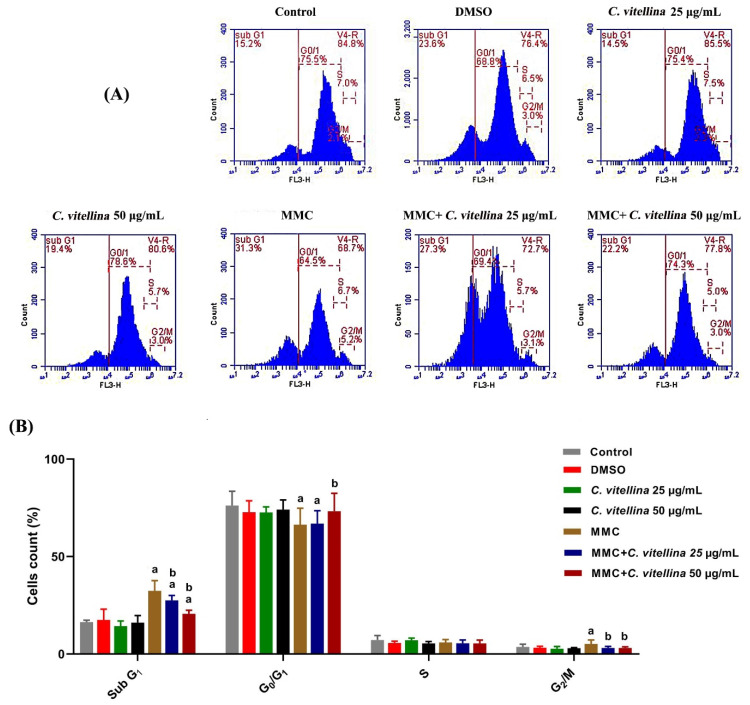
Representative graphs showing the ameliorative effect of *C. vitellina* extract on HPBL cell cycle distribution against MMC-induced alterations, 24 h, using PI staining (**A**). The percentages of cells’ distribution among cell cycle phases are illustrated in the histograms (**B**). The statistical differences (*p* ≤ 0.05), compared to untreated control cells (a) and to MMC-treated groups (b). Data were represented as (Mean ± SD) of three different experiments (n = 3). MMC: Mitomycin C (0.5 µg/mL).

In order to assess the protective effect of the extract on the MMC-altered cell cycle distribution, flow cytometric analyses were performed (Figure 4A). The results showed a significant (*p* ≤ 0.05) apoptotic DNA/sub-G_1_ accumulations in MMC-treated cells when compared with the untreated group. However, co-treatment with both concentrations of the extract (25 and 50 µg/mL) ameliorated the apoptotic effect of MMC, whereas DNA/sub-G_1_ (apoptotic) populations were reduced up to 15.5% and 37% than the MMC-treated cells. Furthermore, MMC caused G_2_/M cell cycle arrest when compared to the untreated cells. While the protective effect was significant (*p* ≤ 0.05) in G_2_/M phase throughout the co-treatment groups with the *C. vitellina* extract, a significant decrease in the events, up to 40% and 42% for 25 and 50 µg/mL of the extract respectively, was recorded when compared to the MMC-treated cells (Figure 4B). The cells’ distribution in G_0/_G_1_ showed a significant reduction in MMC and 25 µg/mL co-treated group when compared to the untreated cells, while the higher concentration of the extract (50 µg/mL) introduced a significant improvement against the MMC toxicity (~10%) (Figure 4B).

### 3.3. Assessment of Genotoxicity

#### 3.3.1. Mitotic Index

**Figure 5:**
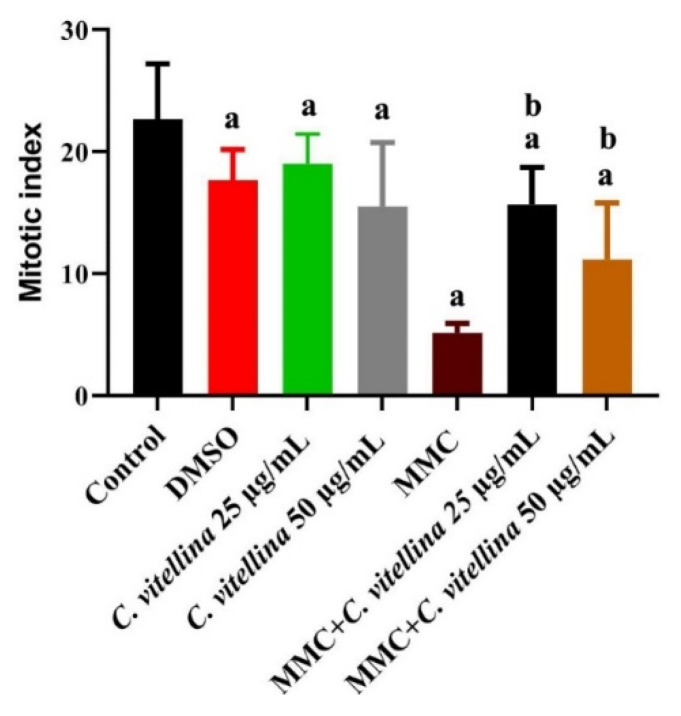
A histogram showing the protective effect of *C. vitellina* extract on HPBL mitotic indexes, 24 h against MMC-induced division arrest, showing significant differences at (*p* ≤ 0.05) with respect to untreated (a) and MMC-treated (b) groups. Data were represented as (Mean ± SD) of three different experiments (n = 3). MMC: Mitomycin C (0.5 µg/mL).

The chromosomal preparation for all treated and control HPBL was performed in order to assess the effect of the treatments on the mitotic index. The exposure to MMC arrested the proliferation along with the diminished mitotic index. Otherwise, the MMC-treated groups combined with 25 and 50 µg/mL of *C. vitellina* revealed a significant (*p* ≤ 0.05) elevation in mitotic index (about 200% and 120%, respectively) when compared to the MMC-treated cells (Figure 5).

#### 3.3.2. DNA Single-Strand Breaks

**Figure 6:**
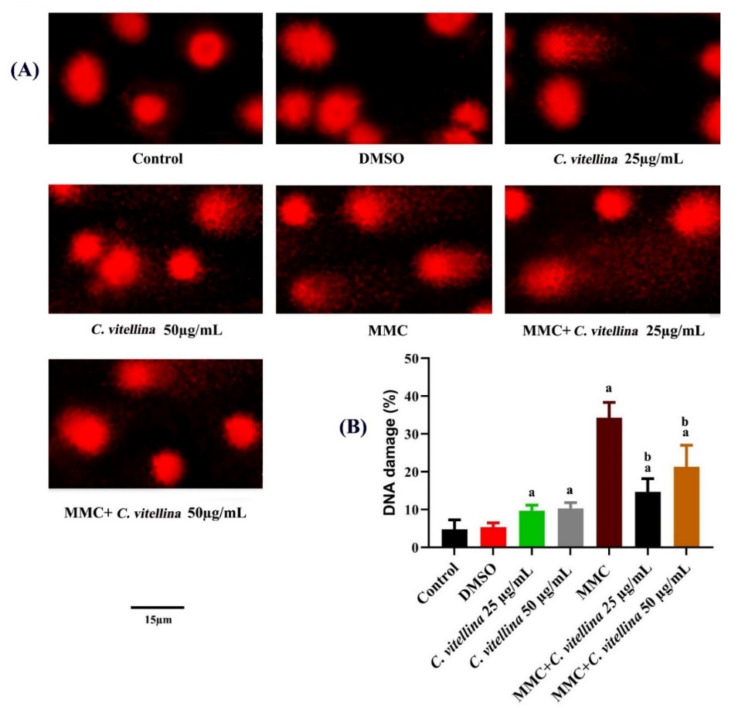
Photomicrographs of alkaline comet assay (**A**) showing the ameliorative effect of *C. vitellina* extract on HPBL DNA-single strand breaks as evaluated by the migration of fragmented DNA after 24 h of treatments against MMC-induced genotoxicity (ethidium bromide staining, Olympus BX 41 fluorescent microscope, Tokyo, Japan). Significant differences at (*p* ≤ 0.05) with respect to untreated and MMC-treated groups are (a) and (b) respectively. Data were represented as (Mean ± SD) of three different experiments (n = 3), (**B**). MMC: Mitomycin C (0.5 µg/mL).

To evaluate the protective effect of *C. vitellina* extract on normal HPBL against the DNA single-strand breaks caused by MMC exposure, single-cell gel electrophoresis (comet assay) was performed in control and treated HPBL (Figure 6A). Results show that MMC-treated cells exhibited significant (*p* ≤ 0.05) DNA damage (34.33 ± 4.04%) with respect to untreated cells. However, the MMC-treated groups combined with 25 and 50 µg/mL of *C. vitellina* revealed a significant (*p* ≤ 0.05) reduction in DNA damage with the percentage of about 57% and 38% respectively, when compared to the MMC-treated group (Figure 6B).

### 3.4. Antihelminthic Activities of C. vitellina Extract

#### 3.4.1. Cytotoxicity of *C. vitellina* on *E. granulosus* Protoscoleces

**Figure 7:**
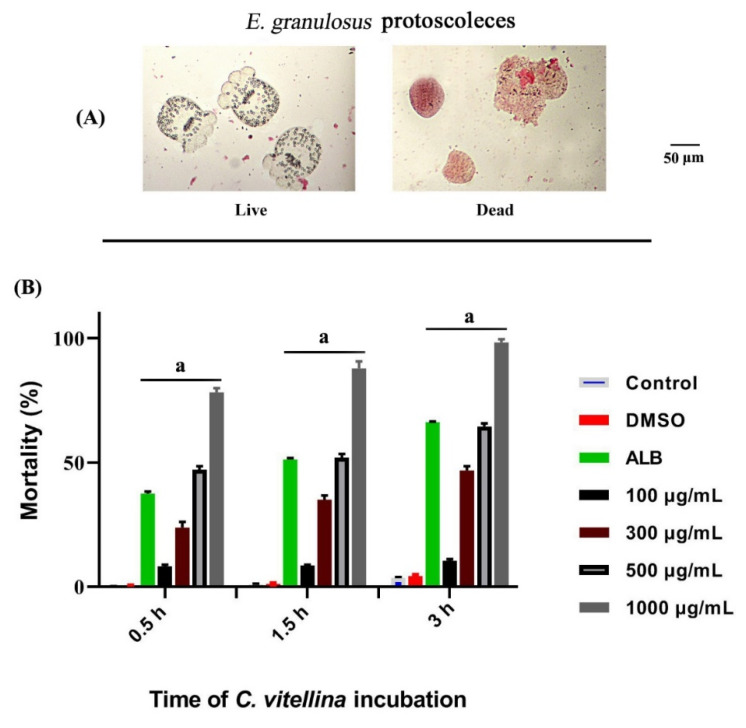
Representative photomicrograph of *E. granulosus* protoscoleces (Olympus BX 41 light microscope, Tokyo, Japan) showing the morphology of alive and dead protoscoleces after eosin staining (**A**). The mean percentage of mortality rates of protoscoleces after the exposure to various concentrations of *C. vitellina* extract for 0.5, 1.5 and 3 h were illustrated in (**B**). Significant differences at (*p* ≤ 0.05) with respect to untreated groups is represented as (a). Data were represented as (Mean ± SD) of three different experiments (n = 3). ALB: albendazole (50 µg/mL).

The current study has investigated the scolicidal effects of *C. vitellina* extract at the concentrations of 100, 300, 500 and 1000 μg/mL compared to 50 µg/mL of albendazole (ALB) as a positive control. The mean percentage of protoscoleces mortality rate after exposure to *C. vitellina* extract at the concentration of 1000 μg/mL at 0.5, 1.5 and 3 h were 78.2%, 87.9% and 98.3%, respectively. However, the mean percentage of mortality rates of protoscoleces after the exposure to the concentration of 500 μg/mL at 0.5, 1.5 and 3 h were 47.1%, 52% and 64.5%, respectively. Furthermore, the exposure to lower concentrations of *C. vitellina* extract of 100 or 300 μg/mL also triggered significant (*p* ≤ 0.05) protoscolicidal effects. These outcomes also confirmed that *C. vitellina* extract at all concentrations had significant (*p* ≤ 0.05) scolicidal effects compared with the untreated groups (Figure 7).

#### 3.4.2. Effect of *C. vitellina* Extract on *E. granulosus* Protoscoleces Ultrastructure

**Figure 8:**
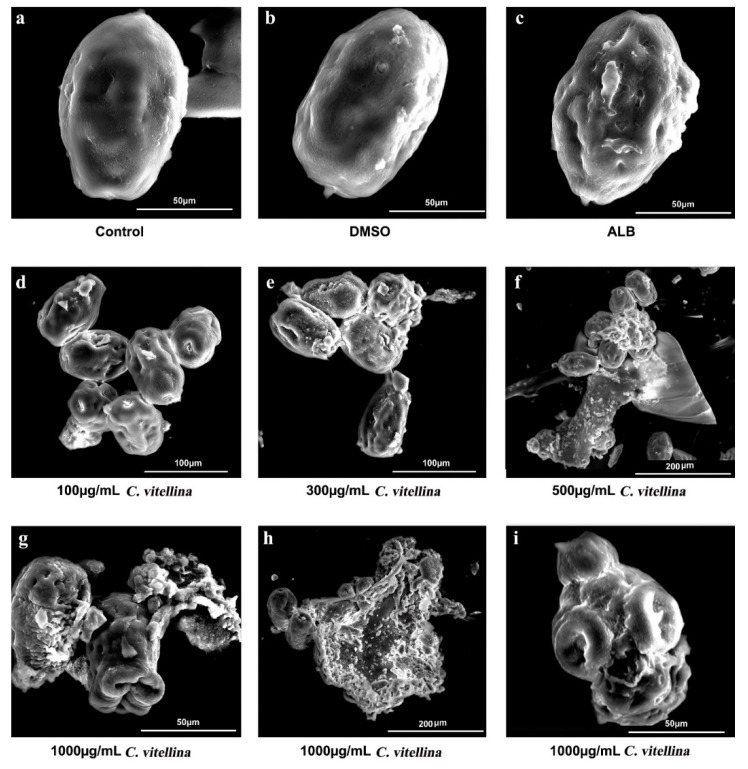
Representative ultramicrographs show the scolicidal effects of *C. vitellina* extract on the morphology of *E. granulosus* protoscoleces after 3 h of the incubation. Where (**a**), control; (**b**), DMSO; (**c**), Albendazole; (**d**), 100 µg/mL; (**e**), 300 µg/mL; (**f**), 500 µg/mL; (**g**–**I**), 1000 µg/mL of the extract. The experiment was done in triplicate (n = 3). ALB: Albendazole (50 µg/mL).

The ultrastructural changes of protoscoleces were evaluated by SEM. The untreated and DMSO-treated groups were represented in Figure 8a,b. The *C. vitellina* extract at a concentration of 100 μg/mL triggered minimal ultrastructural changes including loss of some hooks, contracted soma and some tegumental extensions (Figure 8d). However, the concentration of 300 μg/mL showed more ultrastructural changes including contracted soma, collapsed scolex, and loss of hooks with blebs in the tegument (Figure 8e). At the concentration of 500 μg/mL, SEM showed more aggravated altered structures with loss of hooks, contracted soma to very small size, degenerated scolex and rostellum, remarkable tegumental damage and loss of integrity of the germinal layer (Figure 8f). Similarly, the highest concentration, 1000 μg/mL caused severe degenerative alterations including rostellar disorganization, invaginated scolex, shedding of microtriches, loss of tegumental integrity with altered germinal layer and appearance of blebs (Figure 8g). Moreover, contracted soma regions with loss of hooks, shedding of microtriches, loss of integrity and severe degenerative changes of the germinal layer with many blebs were also noticed (Figure 8h). Figure 8i also supported the effect of *C. vitellina* extract at a concentration of 1000 μg/mL with loss of hooks, shedding of microtriches and contracted soma.

## 4. Discussion

The Japanese originated lichen, *Candelariella vitellina,* is a promising lichen that has previously exhibited significant therapeutic capabilities [54,55], which encouraged further investigations to explore additional biological activities of this pharmaceutically important species. Chemical analysis of the hydromethanolic extract (80%) of *C. vitellina*, using GC-MS and qualitative HPLC, revealed its richness with primary and secondary metabolites such as sugars, alcohols, different phenolic acids, and light flavonoids. It is suggested that the type of phenolics is a catechol phenolic derivative depending on the UV-spectra that exhibited quasi-bands at λ_max_ 211, 234 and 287 nm [73]. The presence of dihydroxy benzoic acid derivatives confirmed the preliminary studies of the ferric chloride test. As well, the light concentration of flavonoid structures confirmed the results of 2D paper chromatography and preliminary studies. On the other hand, the antioxidant activity of *C. vitellina* extract was investigated using the stable radical DPPH model [62]. Results revealed that the good radical-scavenging activities of the extract. These activities may be attributed to the richness of this extract with high phenolic contents with hydroxyl groups that exhibited several mechanisms of antioxidant activity such as radical scavenging and metal ion chelation ability [74]. The majority of identified phenolic acids were previously reported as antioxidants including gallic, chlorogenic, syringic, pyrocatechol, protocatechuic [75], caffeic [76] and ellagic [77]. These compounds have the ability to reduce the development of several diseases [75,78]. Rutin and taxifolin, in the extract, are also considered as strong radical scavengers [79,80]. The acids of primary metabolites such as lactic, acetic, lauric, myristic, palmitic and stearic [81,82,83,84,85] exhibited significant antioxidant activities. Furthermore, soluble sugars in the extract can participate in plant stress response as in vacuolar antioxidant processes [86]. Generally, phenolic compounds are considered the most abundant structures that showed antioxidant activities depending upon the arrangement of functional groups in their structures (i.e., configuration, substitution, and the number of hydroxyl groups) [74,87]. Consequently, many literature studies showed a positive correlation between the number of phenolic compounds and the DPPH free radical scavenging effect [88]. Other antioxidant compounds, in a previous study, such as the polyketide, 3-*O*-(α-d-Ribofuranosyl)-questin, and the terpene, ceriporic acid B were reported in *C. vitellina* extract [54]. In the current study, evaluating the cytotoxicity of *C. vitellina* extract against normal human peripheral lymphocytes (HPBL) using MTT assay resulted in very low cytotoxic impact (IC_50_ > 1000 µg/mL) towards normal HPBL. These may be due to its richness with phenolic compounds that possess antioxidant potentials [89,90]. The antigenotoxic potential of the extract (25 and 50 µg/mL) was also assessed in HPBL against MMC. The observed amelioration of MMC-induced toxicity may be attributed to the antioxidant properties of the extract metabolites [91]. Metabolites, such as ellagic, gallic and caffeic acids, which were detected in the extract, were reported to exhibit in vitro anti-apoptotic effects via a Bcl-2 independent mechanism [89]. Moreover, the free radical scavenging properties of the metabolites such as caffeic and ellagic acids [89] could be the core of the protective potentials against MMC-induced stress, which leads to oxidative DNA damage and apoptosis [31]. The protection by ellagic acid, present in the extract, against nucleosomal and chromosomal damage associated with antioxidative activities was evidenced [92], which may be due to the scavenging potential of the generated free radicals. Moreover, Sevgi et al. [93] demonstrated the protective effect of syringic acid on DNA damage, which may be attributed to its proven antioxidant potentials. The in vivo chromosomal damage induced by gamma-radiation was diminished by chlorogenic acid [94] that may be involved in the antigenotoxic properties exhibited by the extract against the MMC-induced DNA damages and mitotic arrest in this study. Generally, the exposure to MMC resulted in DNA single-strand breaks. The oxidative stress induced by some chemicals usually leads to genetic damages including DNA single or double-strand breaks [32]. Caffeic acid exerted anti-apoptotic and protective activities against the H_2_O_2_-oxidative stress via a Bcl-2 independent mechanism in normal human lymphocytes culture [89]. It protected against the cellular and genetic damages induced by radiation [95]. The antioxidant effect and the ability to inhibit the DNA damage was reported earlier as well [93]. Moreover, the in vitro protective effect of chlorogenic acid against lymphocytes’ genotoxicity via the reduction of oxidative status induced by toxic materials was reported [96]. The action mechanism beyond the antigenotoxic activity of these lichen extract metabolites is still not completely understood, but it is always related to the antioxidant properties of these compounds. In all previously investigated lichens for their antigenotoxicity, strong antioxidant activity was similarly reported [12,97].

The in vitro antihelminthic activities of *C. vitellina* extract against the parasitic platyhelminth, *Echinococcus granulosus* protoscoleces revealed the dose and time-dependent scolicidal effects of *C. vitellina* extract. The observed ultrastructural changes appeared in accordance with Julia and Andrea [98], who studied the effects of metformin and albendazole sulfoxide on protoscoleces of *Echinococcus granulosus*. Furthermore, Verma et al. [99] showed similar ultrastructural alterations while investigating the anticestodal activity of *Endophytic pestalotiopsis* sp. on protoscoleces of hydatid cyst *E. granulosus*. The promising scolicidal effect of the *C. vitellina* extract can be attributed to its richness with active metabolites, such as phenolic compounds that act as defense mechanisms against microorganisms including parasites [100]. Furthermore, gallic acid, catechin, caffeic acid and quercetin were found to have major therapeutic scolicidal effects on hydatid cysts [101,102].

## 5. Conclusions

Our study highlighted the promising antioxidant, antigenotoxic, DNA protective, and antihelminthic capabilities of the Japanese originated lichen, *C. vitellina*, which may be attributed to its richness in bioactive metabolites. Further studies are required to discover lichen’s additional biological activities and therapeutic potentials.

## Figures and Tables

**Table 1:** Phenolic constituents of the *C. vitellina* extract.

Test	Observed Color	Conclusion
Ferric chloride FeCl_3_ (1%)	Intense green (+ve)	A major presence of phenolics
Shinoda’s (Mg/conc. HCl)	Light red (+ve)	Light presence of flavonoids and/or their glycosides
Potassium Iodate (KIO_3_)	−ve	Absent of gallotannins
Sodium nitrite (NaNO_2_)	−ve	Absent of ellagitannins

+ve: positive, −ve: negative.

**Table 2:** Identified compounds of silylated metabolites of *C. vitellina* extract using GC-MS.

Category	Compound Name	Molecular Weight	Molecular Formula	R*_t_* (min)
Acids	2-hydroxy Propanoic acid (D-Lactic Acid)	90	C_3_H_6_O_3_	4.14
	Acetic acid	152	C_8_H_8_O_3_	4.48
	Dodecanoic acid (Lauric acid)	200	C_12_H_24_O_2_	15.04
	3,4 dihydroxy Benzoic acid (Protocatechuic acid)	154	C_7_H_6_O_4_	17.51
	Tetradecanoic acid (Myristic acid)	228	C₁₄H₂₈O₂	18.08
	Hexadecanoic acid (Palmitic acid)	256	C_16_H_32_O_2_	20.85
	Octadecanoic acid (Stearic Acid)	284	C_18_H_36_O_2_	23.43
Alcohols	Glycerol	92	C_3_H_8_O_3_	7.99
	Butane-2,3-diol	90	C_4_H_10_O_2_	12.58
	Ethane-1,2-diol (Ethylene glycol)	62	C_2_H_6_O_2_	12.94
Sugars	Arabinofuranose	150	C_5_H_10_O_5_	13.02
	Xylonic acid	166	C_5_H_10_O_6_	14.37
	D-(+)-Ribono-1,4-lactone	148	C_5_H_8_O_5_	14.53
	α-Xylopyranose	150	C_5_H_10_O_5_	14.84–15.13
	Xylitol	152	C₅H₁₂O₅	15.35
	Methyl-α-D-galactopyranoside	194	C_7_H_14_O_6_	17.75
	D-glucose	180	C_6_H_12_O_6_	18.16
	α-L-Arabinopyranose	150	C_5_H_10_O_5_	18.35
	Methyl-α-D-glucopyranoside	194	C_7_H_14_O_6_	18.41
	Erythritol	122	C_4_H_10_O_4_	18.59
	Ethyl-α-D-galactofuranoside	208	C_8_H_16_O_6_	19.24
Total identified % is 71.23 and SI ≥ 700				

**Table 3:** Identified compounds of *C. vitellina* extract using HPLC.

No.	Identified Metabolites	R*_t_* (min)	Area	Conc. (µg/g)
1	Gallic acid	3.309	1094.90	3160.53
2	Chlorogenic acid	4.105	1513.29	4395.52
3	Caffeic acid	5.740	905.23	1209.61
4	Syringic acid	6.505	580.31	767.17
5	Pyro catechol	6.814	28.25	100.60
6	Rutin	7.324	133.15	684.75
7	Ellagic acid	8.103	32.20	70.18
8	Taxifolin	12.455	79.71	261.14
